# Morphological and molecular characterization of *Crassicauda anthonyi* in Cuvier’s beaked whales from the Canary Islands

**DOI:** 10.1186/s12917-025-04585-3

**Published:** 2025-03-06

**Authors:** Z. Suárez-González, A. Fernández, A. Colom-Rivero, N. García-Álvarez, J. F. González, E. Sierra

**Affiliations:** 1 Veterinary Histology and Pathology, Atlantic Center for Cetacean Research, Veterinary School, University Institute for Animal Health and Food Safety (IUSA), University of Las Palmas de Gran Canaria (ULPGC), Transmontaña S/N, Arucas, Canary Island 35413 Spain; 2 Veterinary School, Buniversity Institute for Animal Health and Food Safety (IUSA), University of Las Palmas de Gran Canaria (ULPGC), Transmontaña S/N, Arucas, Canary Island 35413 Spain

**Keywords:** Cuvier’s beaked whale, *Crassicauda anthonyi*, Nematoda, Canary Island, *ITS2*, *COX1*

## Abstract

Cuvier's beaked whale (CBW) (*Ziphius cavirostris*) is a cosmopolitan species known for its remarkable diving capabilities and is widely distributed across most seas and oceans, including the waters surrounding the Canary Islands. This species frequently exhibits a high prevalence of parasitism by the nematode *Crassicauda* spp., which affects the kidney, urinary tract, and arterial walls via larval migration, and is considered one of the primary natural causes of mortality among CBWs in the Canary archipelago. Despite its significance, molecular identification of this parasite in the Canary Islands has not been systematically conducted until now. To address this gap, 51 CBW’s stranded in the Canary Islands between 1999 and 2023 were necropsied, and 11 adult nematodes were collected from the kidneys of 11 CBWs. Morphological identification and molecular analyses targeting the mitochondrial *COX1* gene and the ITS-2 region were performed, with phylogenetic relationships within the genus *Crassicauda* assessed. Adult parasitism was observed in 86% of the examined animals. Morphological examination identified the species as *Crassicauda cf. anthonyi*, which was corroborated by molecular analyses as *Crassicauda anthonyi*. Renal nodular lesions associated with parasitism were characterized by granulomatous inflammation of varying severity, with intralesional nematodes, fibrosis, necrosis, and multifocal mineralization. *Crassicauda anthonyi* demonstrates a high prevalence and host specificity, contributing to significant renal pathology in stranded CBWs. This study provides parasite morphological data and molecular identification of *C. anthonyi* in the Canary Islands, enhances understanding of its prevalence, and expands both the taxonomy and genetic sequence database for the *Crassicauda* genus.

## Introduction

Nematodes of the genus *Crassicauda* Leiper & Atkinson, 1914 (Order Spirurida) are highly ubiquitous and pathogenic in cetaceans. To date, fourteen different species of *Crassicauda* have been described. These nematodes parasitize a wide range of cetaceans, including both odontocetes and mysticetes [1–5]. *Crassicauda* species have been reported in various organs, such as the genitourinary system, subcutaneous tissue, pterygoid sacs and circulatory system, and are associated with lesions in the affected organs [2, 4, 6, 7]. Certain *Crassicauda* species demonstrate a preference for specific cetacean hosts. For instance, *C. grampicola* exhibits a higher specificity for Risso's dolphin (*Grampus griseus*), infecting the pterygoid sacs, vestibulocochlear nerve, and skull [8–10]. *Crassicauda boopis* is predominantly found in the kidneys of fin whales (*Balaenoptera physalus*) [3, 6, 11, 12], while *C. anthonyi* is primarily associated with the kidneys of Cuvier’s beaked whales (CBWs) (*Ziphius cavirostris*) [13–17]. Among these species, *C. boopis* and *C. anthonyi* are considered particularly pathogenic due to their large size and the migration of their larvae through the circulatory system, which leads to extensive tissue damage [4, 12].

All *Crassicauda* species likely share a similar life cycle. Adult nematodes are thought to excrete eggs via the host’s urine. The eggs are subsequently ingested by intermediate hosts, potentially including cephalopods, that harbor the infective larvae for the final cetacean hosts. However, the possibility of transplacental transmission cannot be ruled out [3, 4, 6, 11, 12, 15, 18].

In histological examinations of mesenteric arteries from CBWs stranded in the Canary Islands, Diaz-Delgado et al. [4] observed nematode larvae in the vascular lumen or in subintimal spaces in affected areas*.* Molecular analysis of two adult nematodes recovered from the kidney of the same CBWs revealed the highest similarity to *C. magna* based on the 18 S ribosomal gene. However, many authors suggest that species identification yields better results when amplifying other genes. In fact, only two species of adult *Crassicauda* have been molecularly identified in CBWs worldwide based on *COX1* and *ITS2*: *Crassicauda giliakiana* and *Crassicauda anthonyi* [3, 6, 14, 15]. Notably, between 1986 and 1998 *C. anthonyi* was morphologically identified in CBWs from the Canary Islands [19]*.* Renal crassicaudiasis in CBWs can induce granulomatous inflammation in the kidneys, leading to varying degrees of renal impairment. In severe cases, these infections can result in chronic kidney disease, disseminated intravascular coagulation, and multi-organ failure [4]. In addition, the larval migration through the blood vessels can cause significant vascular lesions, further compromising the health of the affected animals [4, 6, 14]. In the Canary Islands, infections by *Crassicauda* sp. linked with serious renal and arterial pathological lesions have been frequently detected in stranded CBWs [4, 20, 21], though the nematodes have not been consistently identified to species level using molecular techniques.

This study presents morphological data and the molecular identification of adult nematodes found in the kidneys of CBWs, along with the construction of phylogenetic trees to clarify the species-level classification and evolutionary relationships of these nematodes.

## Material and methods

### Stranding epidemiology data, post-mortem and histopathology examination

Standard necropsies were performed on fifty-one CBWs stranded in the Canary Islands between 1999 and 2023, following established and standardized protocols [20–22]. Necropsies are routinely performed by the veterinary staff on stranded dead animals on the coasts of the Canary Islands and are diagnosed by routine pathological and cause-of-death analyses of stranded cetaceans, performed at the Division of Histology and Animal Pathology of the Instituto de Sanidad Animal y Seguridad Alimentaria (IUSA), within the Faculty of Veterinary Medicine of the Universidad de Las Palmas de Gran Canaria, Spain. Biological data relating to stranding (stranding type and locality) and species (species identification, age category, sex, and body condition) were systematically collected. The locality of each stranding was represented on a geographical map [23]. Age categorization was determined based on total body length and the macroscopic and histological development of the gonads, classifying individuals as fetus, neonate, calf, subadult/juvenile, or adult. Body condition was assessed into several categories (good, moderate, poor, and very poor) based on anatomical parameters. These included the prominence of spinous and transverse processes of the vertebrae, the visibility of the ribs, the mass of the epaxial musculature, and the width of fat deposits [24]. Five codes of conservation condition were established: Code 1 (extremely fresh carcass, as an animal that has recently died or been euthanized), Code 2 (fresh carcass), Code 3 (moderate decomposition), Code 4 (advanced decomposition), and Code 5 (mummified or skeletal remains) [20–22]. Kidney parasite burden was assessed based on adult nematode counts, where fewer than 10 individuals indicated a mild grade, 10–20 a moderate grade, and more than 20 a severe grade.

During the necropsy, all kidneys were thoroughly examined and photographed. Representative samples were collected for subsequent histopathological analysis. Tissue samples fixed in formalin were sectioned and processed using routine procedures, embedded in paraffin, and sectioned at 5 μm. The sections were stained with haematoxylin–eosin (HE) for examination under a light microscope (Olympus BX51, Tokyo, Japan), and a digital camera (Olympus DP21, Tokyo, Japan) was used for imaging. The material used in this study is deposited in the Marine Mammal Tissue Bank, maintained by the Division of Histology and Animal Pathology at the Instituto de Sanidad Animal y Seguridad Alimentaria (IUSA), within the Veterinary School at the Universidad de Las Palmas de Gran Canaria, Spain.

### Morphological characterization of adult nematodes

For morphological analysis, fourteen adult nematodes collected from the kidneys of eleven CBWs (CET593, CET620, CET624, CET645, CET719, CET720, CET770, CET914, CET1242, CET1256, CET1268) were analyzed (Table 1). One adult parasite was selected from each animal, except for CET1268, from which three additional nematodes were analyzed. Since parasites were not systematically collected during necropsy, no nematodes from the remaining animals were available for examination. The nematodes were preserved in 70% alcohol, cleared using a lactophenol solution, and examined on a slide. The caudal portion of fourteen nematodes were obtained and measured, of which 8 were males and 6 were females. For detailed observation, a stereo microscope (Motic SMZ-161) and an optical microscope (Motic BA310E) equipped with a monitor were employed. Motic®Images Plus 2.0 ML software and a Moticam digital camera were used to capture images and measure parasites in mm and μm. Identification of the nematodes was based on measurements and taxonomic keys [11, 25]. The parasite specimens were stored and conserved in 70% ethanol and are deposited in the Marine Mammal Tissue Bank, maintained by the Division of Histology and Animal Pathology at the Instituto de Sanidad Animal y Seguridad Alimentaria (IUSA), within the Veterinary School at the Universidad de Las Palmas de Gran Canaria, Spain.

**Table 1:** Life history data of stranded Cuvier’s beaked whales (*Ziphius cavirostris)* and parasitological analysis (morphological and molecular)

CaseNo	ID Code	Sex	Age	BC	Locality	CC	PGO	MA	PGME
1	CET86	F	A	Good	TF	4	Yes	No	No
2	CET103	M	S/J	Good	FV	3	Yes	No	No
3	CET108	F	A	Good	TF	4	Yes	No	No
4	CET113	F	S/J	Poor	TF	3	No	No	No
5	CET181	M	S/J	ND	FV	2	Yes	No	No
6	CET182	M	S/J	ND	FV	2	Yes	No	No
7	CET183	M	S/J	ND	FV	2	Yes	No	No
8	CET184	M	S/J	ND	FV	2	Yes	No	No
9	CET186	M	S/J	ND	FV	3	Yes	No	No
10	CET187	M	A	ND	LZ	4	Yes	No	No
11	CET188	M	A	ND	LZ	4	Yes	No	No
12	CET189	F	A	ND	FV	5	Yes	No	No
13	CET208	F	S/J	Good	LG	4	Yes	No	No
14	CET236	F	C	Good	LGra	3	No	No	No
15	CET263	M	S/J	Poor	LZ	5	Yes	No	No
16	CET264	F	A	Good	FV	5	Yes	No	No
17	CET265	M	A	Good	FV	5	Yes	No	No
18	CET294	F	A	Good	FV	5	Yes	No	No
19	CET304	F	C	Poor	FV	4	No	No	No
20	CET322	M	A	ND	GC	5	No	No	No
21	CET352	ND	S/J	Poor	TF	4	No	No	No
22	CET471*	F	S/J	Good	FV	2	Yes	No	No
23	CET503*	F	A	ND	GC	5	No	No	No
24	CET576*	F	A	Good	LZ	2	Yes	No	No
25	CET579*	M	S/J	Moderate	TF	4	Yes	No	No
26	CET591*	F	A	ND	TF	4	Yes	No	No
27	CET593*	M	A	Good	GC	5	Yes	CA593	CA593
28	CET620*	M	A	ND	GC	5	Yes	CA620	CA620
29	CET624*	F	A	Good	LGra	3	Yes	CA624	CA624
30	CET645*	M	S/J	Fair	LZ	3	Yes	CA645	CA645
31	CET646*	M	A	Good	FV	4	Yes	No	No
32	CET680	F	N	Fair	GC	5	No	No	No
33	CET688*	F	A	ND	GC	5	Yes	No	No
34	CET712*	F	S/J	Good	FV	5	Yes	No	No
35	CET719*	F	A	Fair	LZ	4	Yes	CA719	CA719
36	CET720	ND	A	ND	FV	5	Yes	CA720	CA720
37	CET770	M	A	Good	TF	3	Yes	CA770	CA770
38	CET771	F	A	Good	TF	2	Yes	No	No
39	CET818	M	S/J	ND	GC	4	Yes	No	No
40	CET833	ND	S/J	Poor	TF	4	Yes	No	No
41	CET855	M	A	ND	GC	4	Yes	No	No
42	CET914	F	S/J	Very Poor	LZ	2	Yes	CA914	CA914
43	CET998	F	A	ND	LZ	4	Yes	No	No
44	CET1027	M	S/J	Fair	TF	2	Yes	No	No
45	CET1048	M	S/J	Poor	GC	2	Yes	No	No
46	CET1074	M	A	Fair	TF	2	Yes	No	No
47	CET1242	F	A	ND	GC	4	Yes	CA1252	CA1252
48	CET1256	F	A	Poor	EH	5	Yes	CA1256	CA1256
49	CET1268	M	A	Good	GC	2	Yes	CA1258	CA1258
50	CET1290	F	A	ND	TF	4	Yes	No	No
51	CET1311	M	S/J	Good	LZ	4	Yes	No	No

Sex: Female (F), Male (M). Age: Adult (A), Subadults/Juveniles (S/J), Calf (C), Neonate (N). Body condition (BC). Locality: Gran Canaria (GC), Tenerife (TF), Fuerteventura (FV), Lanzarote (LZ), La Graciosa (LGr), El Hierro (EH). Conservation Code (CC): Code 1 (extremely fresh carcass), Code 2 (fresh carcass), Code 3 (moderate decomposition), Code 4 (advanced decomposition), and Code 5 (mummified or skeletal remains). Not determined (ND). Parasite grossly observed during necropsy (PGO). Morphological analysis of collected parasites during necropsy (MA). Parasitic genetic material extracted for molecular analysis (PGME). ^*^Animals analyzed in Diaz-Delgado et al. [4]

### Molecular and phylogenic analysis

Eleven adult parasites, one from each CBW -distinct from those used for morphological analysis- were selected for DNA extraction. The specimens consisted of five females and six males, which had been previously preserved in 70% ethanol. A simple heat-alkaline (NaOH) method was employed for the extraction. Specifically, a 1 cm section was excised from each parasite (including cephalic, middle, and caudal regions) and processed in triplicate, adhering to the protocol by [15, 26]. Each section was placed in 1 mL of 50 mM NaOH and heated at 95 °C for 1 h. Following this, the samples were centrifugated at 15,000 rpm for 5 min, and 100 μL of the supernatant was collected, which was then used directly as a template for PCR, following methods outlined by Kumagai et al. [15]. DNA was successfully extracted from all parasite sections.

PCR amplification employed primers targeting the mitochondrial cytochrome c oxidase subunit 1 (*COX1*) gene. The specific primers used were *COX1*-F (JB3); 5′–TTTTTTGGGCATCCTGAGGTTTAT–3′ and *COX1*-R (JB4.5); 5′–TAAAGAAAGAACATAATGAAAATG–3′). Additionally, primers designed for the internal transcribed spacer 2 (*ITS2*) region of ribosomal DNA were also used, namely crassicauda-5.8S-F; 5′–TACTCTTAGCGGTGGATCAC–3′ and crassicauda-28S-R; 5′–AATCACGACTGAGCTGAGGT–3′) [3].

Amplification of *COX1* and *ITS2* regions was carried out according to previously published protocols [3, 15], with adaptations to fit our laboratory conditions. Briefly, for *COX1* amplification, DNA templates (2 μL) were combined in a reaction mixture containing 1.25 mM of each buffer (10 × and MgCl2), 0.25 μM of each PCR primer, 0.25 mM deoxynucleotide triphosphate, and 0.125 U/μL of Taq DNA polymerase (Roche Applied Science), and diethylpyrocarbonate (DEPC)-treated water, reaching a total reaction volume of 10.5 μL. For the amplification of the *ITS2* region, DNA templates (4 μL) were used in a reaction mixture comprising 0.5 mM of each buffer (10 × and MgCl2), 0.5 μM of each PCR primer, 0.5 mM deoxynucleotide triphosphate, 0.25 U/μL of Taq DNA polymerase (Roche Applied Science), and DEPC-treated water to a total reaction volume of 21 μL.

Both *COX1* and *ITS2* PCRs were performed under identical thermal cycling conditions: an initial denaturation at 94º for 5 min, followed by 40 cycles consisting of denaturation at 94ºC for 40 s, annealing at 50ºC for 30 s, and strand extension at 72 ºC for 30 s. After the cycling program, the reaction mixtures were incubated at 72º for an additional 5 min. Genetic material from a parasitized kidney tissue sample from a previously tested animal (animal no. 770) was used as a positive control. Both positive and negative controls were included in the amplification process for each gene. Horizontal gel electrophoresis was performed using 2% agarose gel containing GelRed® (Biotium, Inc., CA, United States) to analyze 5 μL of the obtained amplicons.

Purification of the PCR products for the *COX1* gene was carried out using the Real Clean Spin kit (REAL®, Durviz, S.L., Valencia, Spain) in preparation for bidirectional sequencing using the Sanger method (Secugen S.L., Madrid, Spain). This involved the use of two aliquots of 10 μL of the purified amplicon (at a concentration of 10 ng/ μL) along with 1 μL (5 μM) of each primer, respectively. A BLAST search (BLAST: Basic Local Alignment Search Tool, 2021) was conducted to confirm the identity of the PCR amplicons by comparing them with similar sequences available in GenBank. Both forward and reverse sequences obtained for each *Crassicauda sp.* (excluding primers) amplicon were aligned using the ClustalW algorithm within MEGA11 software (Pennsylvania, PA, United States) [27, 28] to generate a consensus sequence of each parasite from its respective animal.

Fort the *ITS2* region, the amplicons were sent to Secugen S.L., Madrid, Spain for sequencing using the Oxford Nanopore technology (ONT) due to the repetitive nature of the sequence. The purified product from the amplification reaction was ligand-tagged with the SQKNBD114.24 kit and sequenced with Oxford Nanopore technology, MinION FLO-MIN114 R10.4.1. The reads were reanalyzed with stringent quality parameters and filtered based on size (800–1100 nt) and quality (q > 20). In all analyzed samples, a single consensus sequence was obtained for each one, except for sample CA770, which exhibited two distinct sequences with read counts of 1902 and 598, respectively, although the consensus sequence with a higher number of reads of 1902 was selected as the most similar to the rest of the consensus sequences by BLAST analysis. The consensus sequences generated using Amplicon Sorter software were compared to the NCBI standard database with BLAST software. Alignment of the consensus sequences was performed using Multialigment software.

Phylogenetic analysis was performed using the partial *COX1* gene and complete *ITS2* gene sequences, employing the Maximum Likelihood (ML) method with 1,000 bootstrap replicates in MEGA 11 [27, 28]. The analysis utilized the best-fitting evolutionary models determined by the software, specifically the Tamura-nei model for the *COX1* gene and Tamura 3-parameter model for the *ITS2* gene. To ensure the reliability of the phylogenetic tree, a member of the same order, *Spirurida*, was included as the root of the tree. Nucleotide sequences for the partial *COX1* gene from four parasite samples (PP905224-PP905227) and for the complete *ITS2* gene from six parasite samples (PP906995-PP907000) have been deposited in GenBank.

## Results

### Stranding epidemiology data, post-mortem and histopathology examination

**Fig. 1:**
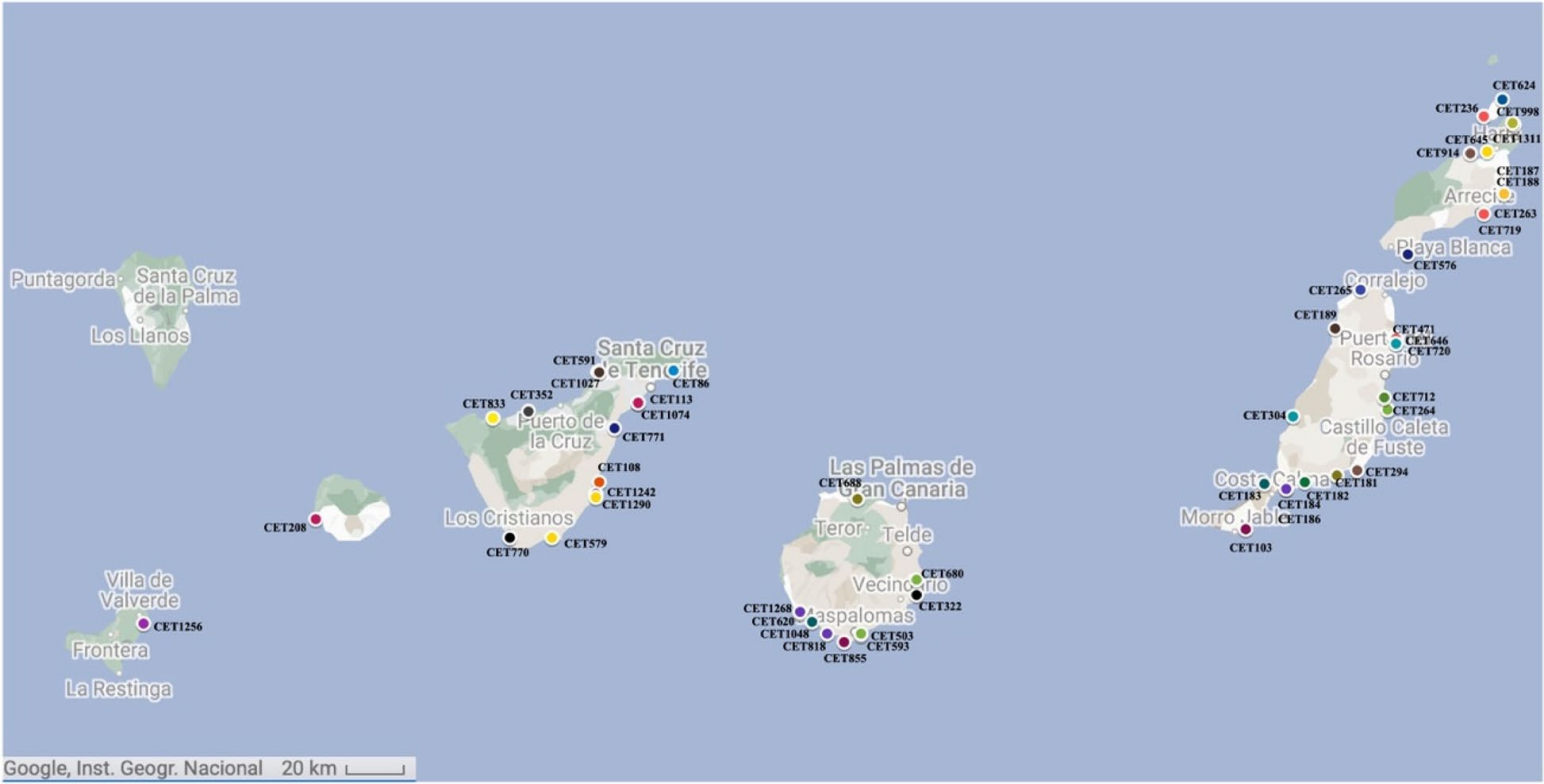
Map of the geographical distribution of Cuvier’s beaked whales stranded in the Canary Islands between 1999 to 2023. Stranding sites for each beaked whale is identified by its case number

Necropsies were performed on fifty-one animals, of which 47% (*n* = 24) were females, 47% (*n* = 24) were males, and in 6% (*n* = 3) the sex could not be determined. Regarding age, 45% (*n* = 23) were adults, 41% (*n* = 21) were subadults/juveniles, 4% (*n* = 2) calves, 2% (*n* = 1) neonates, and 8% (*n* = 4) had an undetermined age. The body condition was categorized as good in 35% (*n* = 18), fair in 12% (*n* = 6), poor in 14% (*n* = 7), very poor in 2% (*n* = 1), and could not be assessed in 37% (*n* = 19). Life history data of stranding are provided in Table 1. The highest number of strandings (35/51) occurred in the eastern islands (Gran Canaria, Fuerteventura, Lanzarote and La Graciosa), compared to the western islands (Tenerife, La Gomera, El Hierro and La Palma) (Fig. 1).

During necropsy, adult nematodes were detected in the kidneys of 86% (44/51) of the examined CBWs, and were collected from eleven individuals for morphological and molecular analysis (Table 1). Nematodes were consistently observed in adult and subadult/juvenile animals (Table 2). In contrast, neonates and calves (3/51) were the only age group of CBWs without evidence of *Crassicauda* sp. infection.

**Table 2:** Age-related prevalence of parasitized Cuvier’s beaked whales (*Ziphius cavirostris*) (*n* = 44)

	Total prevalence of parasitized animals by age (*n* = 44)
Adults	24/44 (55%)
Subadults/Juveniles	20/44 (45%)
Calfs	0/2
Neonates	0/1

**Fig. 2:**
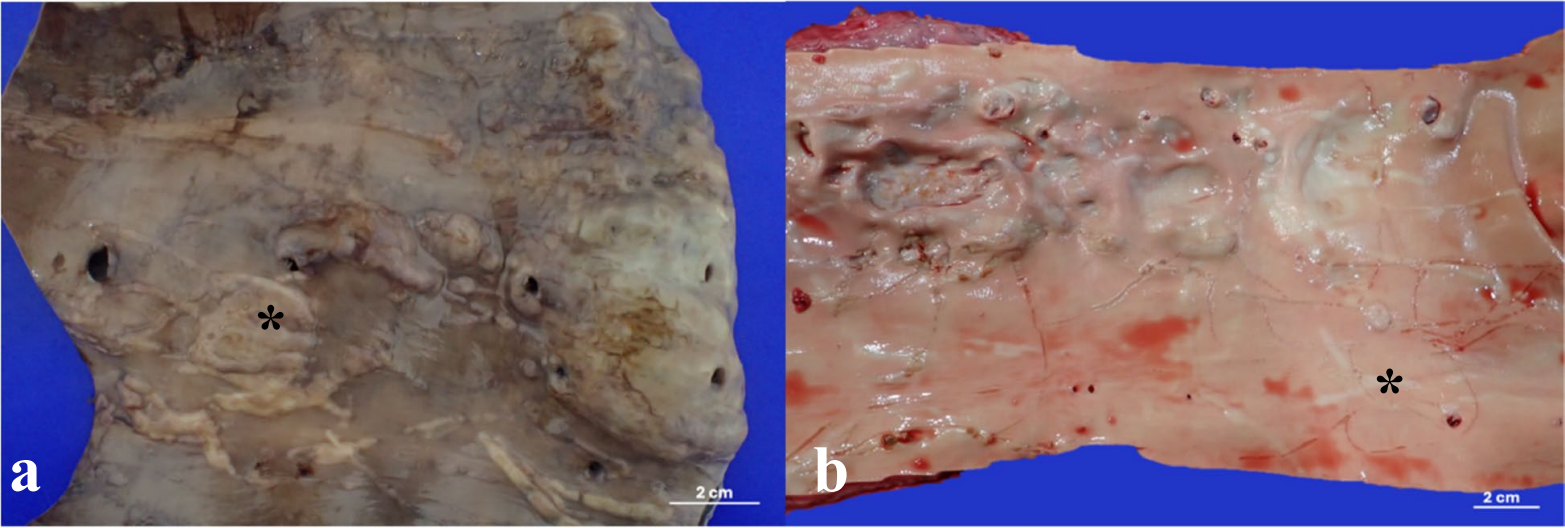
Macroscopic findings in the abdominal and thoracic aorta during necropsy (**a**–**b**). **a** Abdominal aorta of CBW CET1027, showing an abundantly multifocal rough surface with localized tracts in the vascular tunica intima. These tracts vary in direction from straight to zigzag-shaped, differing in size and shape, and forming large, raised yellowish-white areas (asterisk). **b** Thoracic aorta of CBW CET1311, exhibiting tortuous endothelial proliferations and yellowish-white tracts (asterisk) compatible with parasitic migration pathways caused by larval forms and overlying areas

No adult parasites were recorded in the kidneys in 8% of the examined animals (4/51). However, in three individuals, the advanced carcass decomposition may have hindered the detection of existing parasites, while in one animal (no. 113), a comprehensive examination was not possible because the kidneys could not be evaluated due to the absence of the posterior third of its body. Nonetheless, significant arterial lesions consistent with larval migration were observed in most of these cases (Fig. 2a and b).

**Fig. 3:**
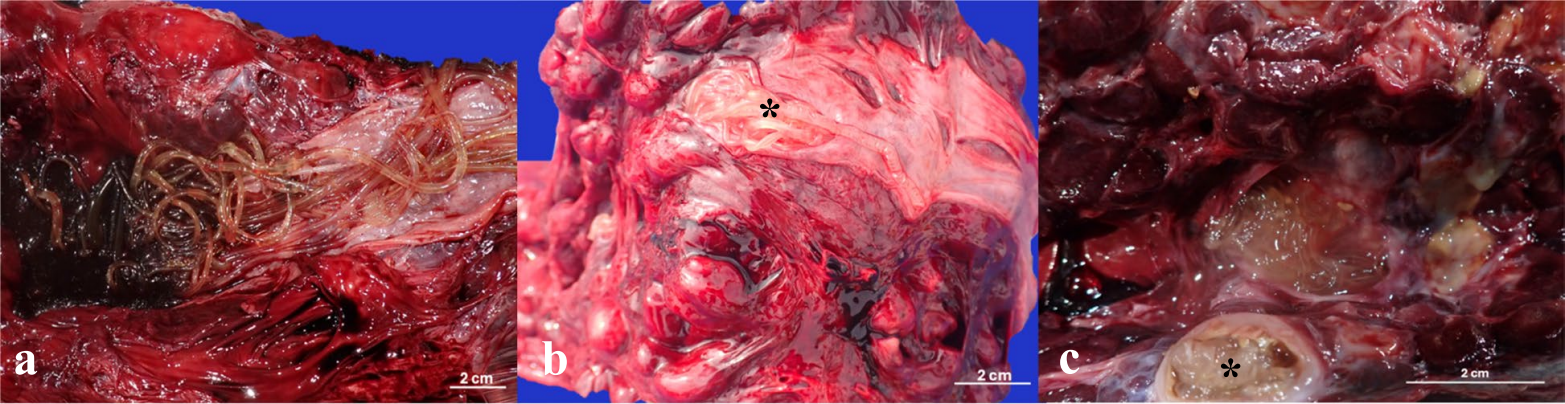
Renal gross findings during necropsy (**a**–**c**). **a** Severe adult nematode parasitism accompanied by interrenal hemorrhages in the left kidney and ureter dilation in CET1268. **b** Moderate presence of adult nematodes in the right ureter (asterisk) of CET1311. **c** Renal granuloma with intralesional nematodes, which completely replace the reniculi (asterisk) in CET720

Among the 44 animals with adult parasites in their kidneys, parasite burden was classified as severe in 52% of cases (*n* = 23), moderate in 43% (*n* = 19), and mild in 5% (*n* = 2). Macroscopically, subcapsular hemorrhages were observed in 9 cases, as well as severe interrenicular hemorrhages in two cases. Adult nematodes were found in the collecting tubules and ureters bilaterally (Fig. 3a and b), along with granulomas that entirely replaced the reniculi, containing intralesional nematodes (Fig. 3c). Additionally, hydroureter and hydronephrosis were observed in two animals (CET103 and CET1268).

**Fig. 4:**
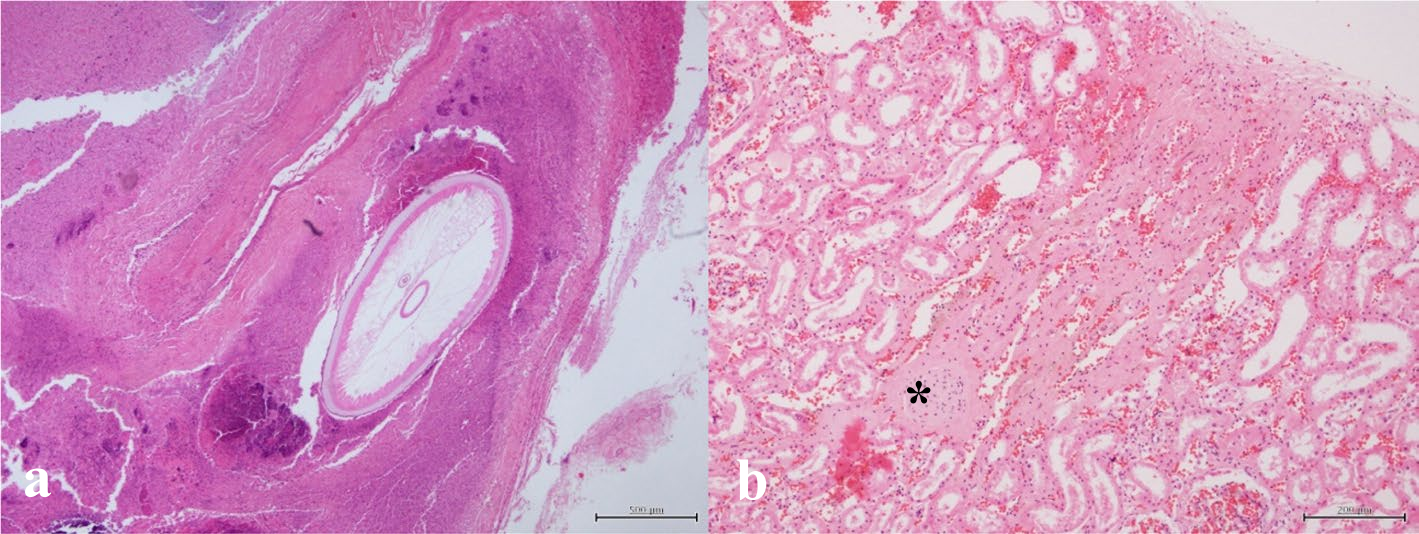
Histopathology of two renal sections (**a**–**b**). **a** Granulomatous inflammatory reaction associated with the presence of intralesional nematodes (multifocal granulomatous interstitial nephritis) in CET1242. HE stain, × 4. **b** Renal cortex showing marked congestion, multifocal hemorrhages, moderate multifocal interstitial fibrosis, and glomerular sclerosis (asterisk) in CET1268. HE stain, × 10

Histopathological examination revealed lesions primarily characterized by granulomatous nephritis of varying severity, with intralesional nematode parasites (Fig. 4a), renicular atrophy, multifocal calcifications, glomerular sclerosis, fibrosis, and hemorrhages (Fig. 4b). Additionally, hyaline cylinders were present in most animals at the medullary level.

#### Morphological characterization of adult nematodes

**Fig. 5:**
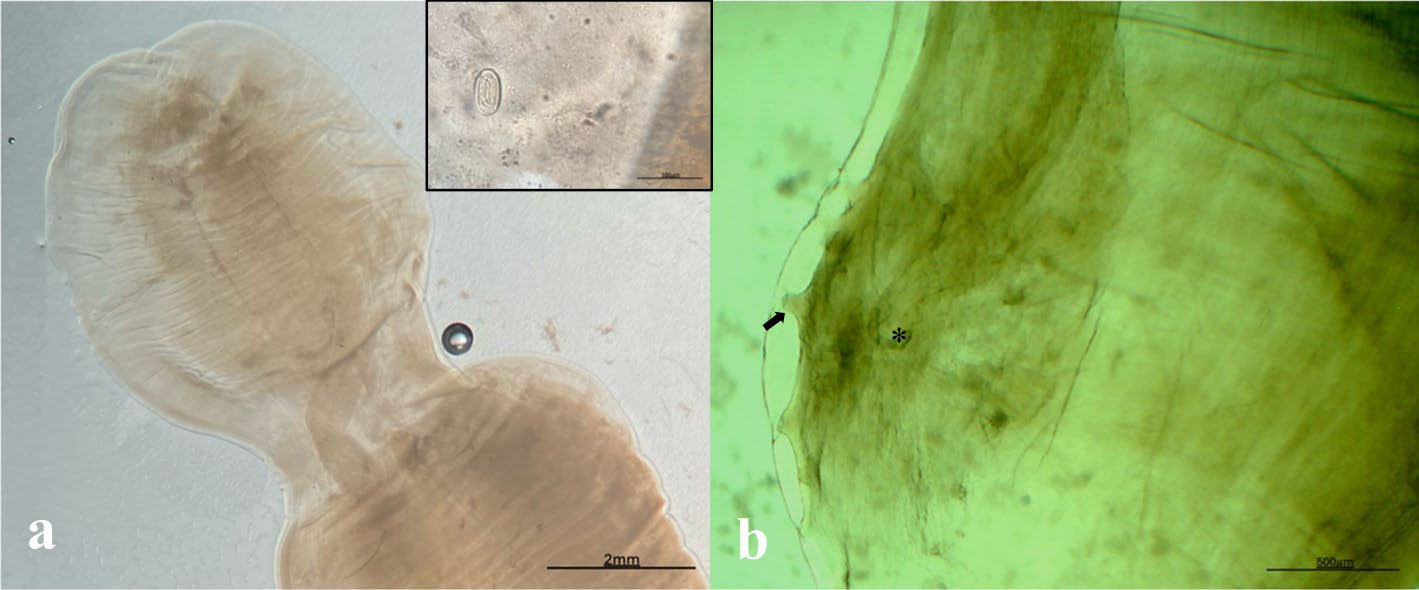
Microscopic view of the caudal end in female and male *Crassicauda* cf. *anthonyi* (**a**-**b**). **a** Microscopic view of the caudal end of a female showing posterior narrowing, × 1.25. Inset: Oval-shaped, larvated egg, × 10. **b** Microscopic view of the caudal end of a male with details of the papillae (arrow) and spicules (asterisk), × 4

Among the CBWs harboring adult kidney nematodes, samples were collected from only eleven individuals. Fourteen adult nematodes were analyzed, one from each CBW, and three additional nematodes from CET1268. Adult nematodes were identified as *Crassicauda* sp. based on the morphologic and morphometric characteristics of the posterior ends [1]. The caudal end of 8 males and 6 females were measured. The total length of neither males nor females could be determined; however, two long fragments measuring 32 cm (from a male) and 64 cm (from a female) were recorded. Females exhibit a narrowing at the caudal end, where the vulva opens posteriorly (Fig. 3a). The total width in females was measured cranial to the constriction at the posterior end, ranging from 3.2 to 4.0 mm. The eggs (*n* = 10) were oval-shaped, contained larvae, and had thick shells, with an average length of 58 µm and an average width of 37 µm. The lengths ranged from 47 to 63.9 µm, and the widths ranged from 35 to 43.9 µm (Inset Fig. 5a). Males characteristically had a spiral or hook-like posterior end with a rounded tip. Width measurements were taken cranial to this hook, ranging from 2.0 to 2.8 mm. The spicules were notably small and uniform in length (216.2–168.2 µm), with a variable number of asymmetrical papillae (Fig. 5b), ranging from 9 to 10 papillae per individual, with 4 to 5 on each side. Based on these morphological characteristics, it is suggested that the species involved is *Crassicauda* cf. *anthonyi*.

### Molecular and phylogenic analysis

DNA extraction was performed in triplicate on three fragments (cephalic, medial, and caudal) from the caudal end of each of the 11 adult nematodes collected from the 11 CBWs. In total, DNA was extracted from 33 fragments. The concentrations of each fragment were measured, and the highest quality fragments from each nematode were selected and subjected to PCR amplification. In addition, samples were subjected to horizontal gel electrophoresis. As a result, DNA amplification was successful in 6 parasites from 6 out of 11 animals (Table 3). For the *COX1* gene, PCR effectively amplified parasites from four animals, yielding sequences of good quality (GenBank accession numbers PP905224- PP905227) with lengths ranging from 375–395 bp (Table 3).

**Table 3:** Data of *Crassicauda anthonyi* nematodes recovered from Cuvier’s beaked whales (*Ziphius cavirostris)* stranded in the Canary Islands

Nematode ID	CET ID	Species name	Sex	Portion sample	*COX1* gene ID	*ITS2* gene ID
CA719	CET 719	*Crassicauda anthonyi*	Female	Cephalic	PP905224 (375 bp)	PP906995 (902 bp)
CA720	CET 720	*Crassicauda anthonyi*	Male	Cephalic	-	PP906996 (895 bp)
CA770	CET 770	*Crassicauda anthonyi*	Female	-	PP905225 (394 bp)	PP906997 (902 bp)
CA914	CET 914	*Crassicauda anthonyi*	Female	Middle	PP905226 (395 bp)	PP906998 (899 bp)
CA1242	CET 1242	*Crassicauda anthonyi*	Female	Cephalic	-	PP906999 (904 bp)
CA1256	CET 1256	*Crassicauda anthonyi*	Female	Cephalic	PP905227 (394 bp)	PP907000 (900 bp)

*bp* base pairs

**Fig. 6:**
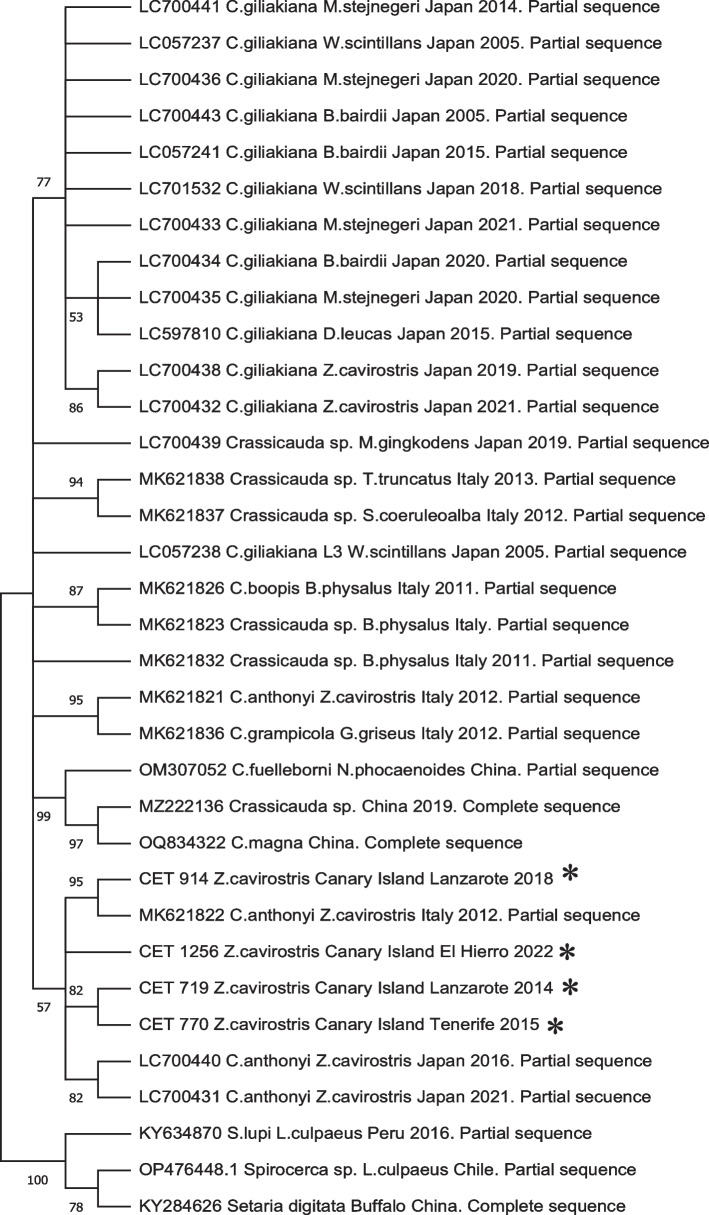
Phylogenetic analysis based on the *COX1* gene. Analysis was performed by MEGA11 using the maximum likelihood method (ML) with 1,000 bootstrap replicates and included *Spirocerca lupi*, *Spirocerca* sp. and *Setaria digitata* as outgroup. *Sequences obtained in this study

**Fig. 7:**
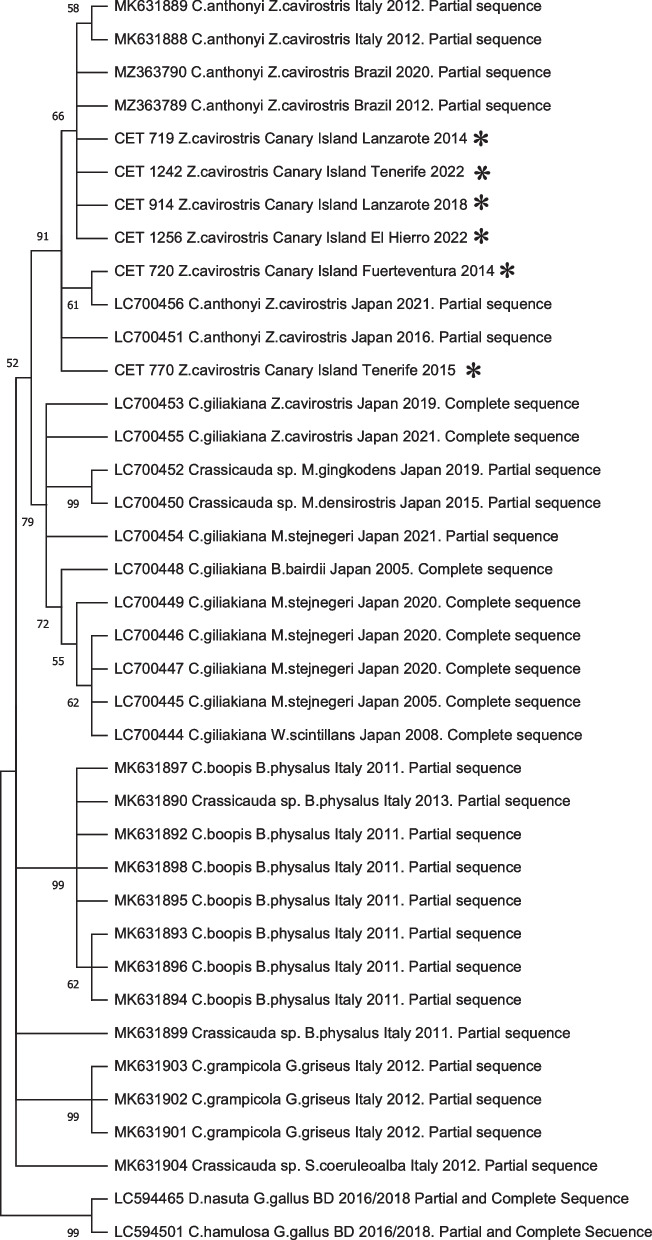
Phylogenetic analysis based on the *ITS2* gene. Analysis was performed by MEGA11 using the maximum likelihood method (ML) with 1,000 bootstrap replicates and included *Dispharynx nasuta* and *Cheilospirura hamulosa* as outgroup. *Sequences obtained in this study

Performing a BLAST search, 96–97% homology was found with other available *C. anthonyi* sequences and with a query cover of 99%. Phylogenetic analysis of the mitochondrial *COX1* gene revealed that the adult renal parasites from CBWs from our study clustered with *C. anthonyi*, consistent with all corresponding sequences in GenBank, except for sequence MK621821 (listed as *C. anthonyi* in GenBank), which grouped within the *C. grampicola* cluster (Fig. 6). For the *ITS2* region, sequences were obtained from 6 parasites from 6 out of 11 animals, with alignment lengths ranging from 895–904 pb (GenBank accession numbers PP906995- PP907000) (Table 3). BLAST analysis of the obtained sequences showed a query cover of 90–99% and a sequence similarity of 92.93–93.6% with *C. giliakiana*, whereas *C. antonyi* exhibited a query cover of 43–61% and a sequence similarity of 99.24–99.64%. This could be explained by the longer sequences than those published. Consequently, the specimens collected from the six animals were identified as *C. anthonyi* based on the *ITS2* region, with this identification further supported by phylogenetic analysis. The resulting phylogeny closely mirrored that of the *COX1* gene analysis (Fig. 7) and was consistent with phylogenetic trees from previous studies. Notably, the bootstrap values obtained through the ML method were higher than those derived from the *COX1* analysis, reinforcing prior findings that the ribosomal DNA region provides greater reliability for phylogenetic tree construction.

## Discussion

Over a 24-year period, 51 Cuvier’s beaked whales were necropsied in the Canary Islands. A consistent finding during necropsies was the presence of large nematodes in the kidneys and urinary tract, identified as *Crassicauda* sp., which have been implicated in renal parasitosis and associated with severe arterial lesions [4, 17, 20, 21]. This pathology has been documented globally in CBWs [3, 13–16], with the first reported case involving a True’s beaked whale (*Mesoplodon mirus*) along the Atlantic coast of France [25]. In this study, evidence of lesions at the targeted site of *Crassicauda* sp. infection, the kidneys, was observed. Macroscopically, the lesions were predominantly characterized by granulomas containing intralesional nematodes, which replaced the reniculi, along with hemorrhages and the presence of nematodes in the collecting tubules and ureters. Microscopically, the findings included granulomatous nephritis with fibrosis, hemorrhages, calcifications, and renal atrophy associated with intralesional nematodes, consistent with previous reports [3, 4, 14, 29]. Notably, no renal parasitism or related lesions were detected in the calves or neonates examined in this study. In contrast, a high prevalence of parasitism was observed in adults and subadult/juvenile individuals. The absence of *Crassicauda sp.* infection in younger animals may reflect the increased risk of infection as animals mature and consume infected cephalopods, putative intermediate hosts for this parasite. However, the small sample size of this age group (*n* = 3) may also account for this finding. Lambertsen [18] previously suggested the possibility of maternal or fetal transmission in the case of *C. boopis*; however, further research with a larger sample size is needed to investigate the potential presence of *Crassicauda* spp. in young CBWs and to evaluate this hypothesis. Additionally, arterial lesions of varying severity were observed in all adult and subadult/juvenile animals harboring adult nematodes in the kidneys. These lesions were not further examined in the present study, as they had already been investigated by Díaz-Delgado et al. [4] in a subset of animals (13/51) included in this study, where larval forms were detected within the arterial lumen or walls in 2 out of 13 cases. It is hypothesized that *Crassicauda sp.* in CBWs follows a life cycle similar to other *Crassicauda spp*. that parasitize the kidney, such as *C. boopis* in mysticetes. In this cycle, larvae may initially reach the intestine, where they form nodules and penetrate the mucosal and submucosal layers, eventually migrating through the mesenteric arteries to the aorta and renal arteries [3, 18]. Notably, the intestines were not examined in any of the studies.

Previous research in the Canary Islands had not focused on identifying the specific species responsible for renal parasitosis in CBWs. For the morphological analysis, 14 caudal ends from nematodes collected from the kidneys of 11 CBWs were examined. In total, 8 caudal ends from adult male nematodes and 6 from adult female nematodes were analyzed. Additionally, egg measurements were averaged. Based on the morphometric characteristics of the nematodes, the species involved was suggested to be *Crassicauda cf. anthonyi*. aligning with existing literature [11, 25]*.* For the molecular analysis, DNA was extracted from 11 nematodes collected from the 11 CBWs in which adult parasites were available. DNA amplification was successful in 4 nematodes for the *COX1* gene and in 6 nematodes for the *ITS2* gene. Agarose gel electrophoresis was performed and the amplified products were sequenced. Consensus sequences for both genes were obtained from 4 parasite samples corresponding to animals CET 719, CET 770, CET 914, and CET 1268. Comparison of our sequences with published data confirmed the molecular identification of the species as *Crassicauda anthonyi*. In the case of the *ITS2* region, BLAST analysis revealed a lower query coverage with other published *C. anthonyi* sequences compared to *C. giliakiana* sequences. However, homology was higher with *C. anthonyi*. This discrepancy may be attributed to the shorter length of previously published *ITS2* sequences (393–549 bp) compared to those obtained in this study (895–904 bp). Phylogenetic analysis further corroborate this findings, as the sequences clustered with the *C. anthonyi* clade, in agreement with the results obtained in previous reports [3, 14, 15]. Notably, phylogenetic analysis using the *ITS2* region yielded a higher bootstrap value when employing the ML model compared to the *COX1* region. Similar observations have been reported in earlier studies [15], suggesting that the *ITS2* region may be more reliable for phylogenetic investigations of the *Crassicauda* genus. However, sequencing the *ITS2* region presented challenges due to its high degree of homogeneity, which made it difficult to obtain complete amplicon sequences using Sanger sequencing. To overcome this limitation, Oxford Nanopore technology was employed as an alternative sequencing approach.

Phylogenetic studies indicate that *C. anthonyi* is a distinct species closely related to *C. giliakiana*. Both species share morphological similarities and inhabit the kidneys of odontocetes [15, 25, 30, 31]. *Crassicauda giliakiana* has been reported in members of the family Ziphiidae, including *Mesoplodon stejnegeri*, *Berardius bairdii*, and *Ziphius cavirostris* [15], as well as in *Delphinapterus leucas* [32]. In contrast, *C. anthonyi* has been documented exclusively in CBWs across various geographic regions [13–16], supporting the hypothesis that *C. anthonyi* exhibits a strong host specificity for CBWs.

The 18S ribosomal gene (amplified using primers 930F and 1200 R) from two adult kidney nematodes in one animal from our study was previously sequenced by Díaz-Delgado et al. [4]. In that study, the closest species identified was *C. magna*. However, previous research has shown that the 18S region is highly conserved among *Crassicauda* species, making it useful for genus-level identification but not for species-level identification [3]. Based on the findings of the present research, one of the parasites responsible for these renal parasitosis in stranded CBWs in the Canary Islands is *C. anthonyi*. To strengthen this conclusion, further research on renal crassicaudiasis caused is essential. Routine species identification in stranded CBWs should be prioritized, along with a more comprehensive examination of the arteries and intestines to detect and characterize larval stages through detailed morphological and molecular analyses. This would improve our understanding of renal and arterial crassicaudiasis in this species, recognizing it as a severe pathology with significant implications for whale health and survival [4].

These results provide morphological data and the molecular identification of the nematodes responsible for renal crassicaudiasis in CBWs in the Canary Islands, molecularly confirming *Crassicauda anthonyi* as the causative species. This study contributes to a better understanding of this parasitosis and expands the available sequence data for the *Crassicauda* genus in genetic database. However, comprehensive studies focusing on the detection and identification of larvae in CBWs in the region are still needed to further enhance knowledge of the parasite’s life cycle.

## Data Availability

All data generated or analyzed during this study are presented in this article. Additionally, the sequences generated in this study are publicly available in the GenBank database. The accession numbers for the COX1 region sequences are as follows: PP905224 (CA719), PP905225 (CA770), PP905226 (CA914), and PP905227 (CA1256). For the ITS2 region sequences, the accession numbers are: PP906995 (CA719), PP906996 (CA720), PP906997 (CA770), PP906998 (CA914), PP906999 (CA1242), and PP907000 (CA1256).
